# Identification of veterinary and medically important blood parasites using contrastive loss-based self-supervised learning

**DOI:** 10.14202/vetworld.2024.2619-2634

**Published:** 2024-11-25

**Authors:** Supasuta Busayakanon, Morakot Kaewthamasorn, Natchapon Pinetsuksai, Teerawat Tongloy, Santhad Chuwongin, Siridech Boonsang, Veerayuth Kittichai

**Affiliations:** 1 Faculty of Medicine, King Mongkut’s Institute of Technology Ladkrabang, Bangkok 10520, Thailand; 2 Department of Pathology, Center of Excellence in Veterinary Parasitology, Faculty of Veterinary Science, Chulalongkorn University, Bangkok 10330, Thailand; 3 College of Advanced Manufacturing Innovation, King Mongkut’s Institute of Technology Ladkrabang, Bangkok 10520, Thailand; 4 Department of Electrical Engineering, School of Engineering, King Mongkut’s Institute of Technology Ladkrabang, Bangkok 10520, Thailand

**Keywords:** bootstrap your own latent, fractioned data, microscopic image, pre-trained, self-supervised learning, zoonotic disease

## Abstract

**Background and Aim:**

Zoonotic diseases caused by various blood parasites are important public health concerns that impact animals and humans worldwide. The traditional method of microscopic examination for parasite diagnosis is labor-intensive, time-consuming, and prone to variability among observers, necessitating highly skilled and experienced personnel. Therefore, an innovative approach is required to enhance the conventional method. This study aimed to develop a self-supervised learning (SSL) approach to identify zoonotic blood parasites from microscopic images, with an initial focus on parasite species classification.

**Materials and Methods:**

We acquired a public dataset featuring microscopic images of Giemsa-stained thin blood films of trypanosomes and other blood parasites, including *Babesia, Leishmania, Plasmodium, Toxoplasma*, and *Trichomonad*, as well as images of both white and red blood cells. The input data were subjected to SSL model training using the Bootstrap Your Own Latent (BYOL) algorithm with Residual Network 50 (ResNet50), ResNet101, and ResNet152 as the backbones. The performance of the proposed SSL model was then compared to that of baseline models.

**Results:**

The proposed BYOL SSL model outperformed supervised learning models across all classes. Among the SSL models, ResNet50 consistently achieved high accuracy, reaching 0.992 in most classes, which aligns well with the patterns observed in the pre-trained uniform manifold approximation and projection representations. Fine-tuned SSL models exhibit high performance, achieving 95% accuracy and a 0.960 area under the curve of the receiver operating characteristics (ROC) curve even when fine-tuned with 1% of the data in the downstream process. Furthermore, 20% of the data for training with SSL models yielded ≥95% in all other statistical metrics, including accuracy, recall, precision, specification, F1 score, and ROC curve. As a result, multi-class classification prediction demonstrated that model performance exceeded 91% for the F1 score, except for the early stage of *Trypanosoma evansi*, which showed an F1 score of 87%. This may be due to the model being exposed to high levels of variation during the developmental stage.

**Conclusion:**

This approach can significantly enhance active surveillance efforts to improve disease control and prevent outbreaks, particularly in resource-limited settings. In addition, SSL addresses significant challenges, such as data variability and the requirement for extensive class labeling, which are common in biology and medical fields.

## Introduction

Zoonotic diseases caused by various blood parasites are a public health concern, impacting animals and humans worldwide. Trypanosomiasis is a well-known disease caused by various blood parasites of the *Trypanosoma* genus [1]. The zoonotic species of the *Trypanosoma* genus, which can infect several hosts across vast geographic regions, significantly impacts livestock production and poses a global health challenge [2–4]. Active surveillance is necessary to control the spread of the disease. In addition to sleeping sickness and Chagas disease, which are caused by *Trypanosoma brucei* and *Trypanosoma cruzi*, respectively, *Trypanosoma lewisi* has also been shown to cause pediatric infections in Thailand [5]. Trypanosomiasis or Surra caused by *Trypanosoma evansi* typically affects animals worldwide and predominantly affects camels, cattle, buffaloes, and horses. This parasite is endemic to tropical regions, including South America, Africa, Eastern Asia, and Southeast Asia [2, 6]. The parasite has recently been reported to infect humans, although its transmission route remains unclear. Typically, it is transmitted to hosts by the bites of tabanid or tsetse flies or accidentally through open wounds [7]. Microscopic examination is the standard diagnostic method [8]. Nevertheless, it is tedious, time-consuming, and requires multiple steps to prepare thin blood films. In addition, this method suffers from intra- and inter-observer variability and requires highly skilled and experienced personnel [9]. Consequently, a novel, innovative approach is required to improve on the traditional method. Trypanosomes have attracted attention due to their potential for zoonotic transmission, as demonstrated by several reports of human cases in South and Southeast Asian countries, such as India and Vietnam [7, 10]. Vietnam and Thailand share environmental similarities, highlighting the relevance of this issue in the region. The symptoms exhibited by patients closely resemble those of sleeping sickness caused by *T. brucei*, a typical human-infecting species [7, 10]. Consequently, this presents a significant risk to the local population, particularly in Thailand, where dogs, racing horses, and water buffaloes in the eastern region have been reported as reservoirs of *T. evansi* [11]. This scenario increases the possibility of parasite transmission from animals to humans in local Thai communities. Traditional diagnostic techniques for several trypanosome parasites include serological, molecular, and microscopic tests [7, 10, 12]. This method uses a thin blood film stained with Giemsa to identify the parasite [13–15]. This traditional approach is preferred due to its affordability, quick sample preparation, and minimal need for sophisticated medical equipment. Nevertheless, it necessitates expertise, skills, and experience for precise diagnosis, which is time-consuming and labor-intensive during the entire process. Furthermore, variations in observations between different technicians and within the same technician can affect the reliability of the assessment [8, 16]. As a result, this method may not be suitable for rapid screening, especially in remote areas where resources and specialized personnel are limited. Recently, deep learning has emerged as a practical technique in the medical field for automating the screening and identification of parasites [17, 18]. Although many current studies rely on supervised learning (SL), which requires extensive labeled training data from experts, this limitation can be overcome using a deep learning model, particularly self-supervised learning (SSL). This method requires smaller portions of labeled data, thereby reducing the burden of labeling and enhancing the performance of downstream tasks [19]. This approach offers an appropriate solution for developing detection tools to complement microscopic examination, especially in scenarios with constraints on labeling and complexity in image interpretation [20, 21]. Deploying such advanced technology would address the aforementioned limitations and streamline surveillance, enabling the swift, effective, and dependable identification of high-risk areas for animal infections that could pose a threat to humans. This approach enhances awareness of this unusual infection among the population and facilitates effective control and treatment of infected animals. Consequently, it is crucial to mitigate potential outbreaks and promote public health in affected regions.

Recently, a tool based on deep learning for parasite detection and classification was developed. This tool strengthens and complements rapid microscopic examination in areas lacking adequate medical facilities and expertise [17]. Most existing research relies on SL methods, necessitating particular annotation work by specialists and several labeled datasets to realize high-performance tools. However, an intriguing study conducted by Ha *et al*. [16] used a different approach to effectively detect and classify apicomplexan parasites (*Plasmodium, Toxoplasma*, and *Babesia*). Ha *et al*. [16] introduced semi-supervised graph learning (SSGL), a novel framework comprising convolutional neural network (CNN) feature embedding, graph learning algorithms, and graph convolutional neural networks (GCNs). Segments of *Plasmodium, Toxoplasma, Babesia*, and red blood cells obtained from publicly available Mendeley (https://www.mendeley.com/) data comprising labeled and unlabeled samples are fed into a CNN model. Residual Network 50 (ResNet50) is employed to extract a data representation and plot each representation as a node. The relationships or edges between nodes are then computed based on similarity using a graph learning algorithm. The process generates an adjacency matrix, establishing correlations among unlabeled and labeled samples. The learnable adjacency matrix is then applied to the GCN to process the graph structure data and perform classification. This study compares the proposed SSGL method to three fully SL approaches proposed by VGG Net [22], Google Net [23], and ResNet50 [24]. Two classical semi-supervised methods, Pi-model and Mix-Match, are also included for comparison. They also varied the amount of labeled data from 20% to 100% for assessment and comparison with other baseline models. The SSGL result is particularly noteworthy because it outperforms other methods. The SSGL approach achieved an accuracy of 91.83% with only 20% label data [16]. Hence, this study highlights the significance of employing methods other than traditional SL, especially when acquiring much labeled datasets is laborious and time-consuming. By exploring techniques like semi-SL, we can fully exploit the capabilities of AI to address complicated tasks such as parasite detection and classification.

Bootstrap Your Own Latent (BYOL) is a self-supervised image representation learning method proposed in 2020 [25]. The proposed method was developed from the contrastive method, which represents state-of-the-art image representation learning. In contrastive methods, the trained image is represented using both positive and negative pairs [19, 25]. The positive pair comprises diverse augmentations of a single image, and the negative pair comprises augmentations of distinct images. The proposed method works by minimizing the distance between positive pairs and maximizing the distance between negative pairs, thereby establishing an optimal condition for effective image representation learning [19]. The negative pair has an advantage relative to preventing the collapse solution, where the encoder produces identical representations for all images, thus failing to learn meaningful features. However, employing negative pairs has several drawbacks. These include the necessity for careful handling of negative pairs, the controversy over which negative pair is better in the learning process, and the increased computational burden required to simultaneously manage both positive and negative pairs. The BYOL offers an innovative solution to address these limitations and prevent collapse. It employs two neural networks, the online network and target network, which learn from each other [25]. Both networks share the same architecture, namely, the ResNet model, except the prediction stage, which is only applicable to the online network. Initially, the weight of the online network is updated, while the weights of the target network are maintained as the exponential moving average of the online network’s parameters. In the architecture of the BYOL, the input image is subjected to augmentation. These augmentations include random rotations, flips, color distortions, and other transformations intended to augment the diversity of the training data [25]. Subsequently, the image in its different augmentations is fed into the encoder f_ꝋ_ of the online network and f_£_ of the target network. The online network processes the augmented images, generating the latent presentation y_ꝋ_ for each input, downsizing the representation (z_ꝋ_), and making a prediction for the target network at the end of the online network pipeline. The contrastive loss, calculated from the mean square error between two networks, is utilized to update the weights of the online network through the backpropagation process, ensuring that the weight of the target network is adjusted accordingly. Contrastive loss plays a crucial role in BYOL because it prompts the online network to produce similar representations for the same image under various augmentations. Simultaneously, it encourages the embeddings of the online network to differ from those of other images. This approach allows the model to learn meaningful and invariant representations from unlabeled data without relying on negative pairs. This iterative process is repeated to minimize loss and refine the model representation [25]. These representations can then undergo fine-tuning for specific downstream tasks using labeled data, rendering the model applicable to various applications. The proposed BYOL approach demonstrated promising outcomes on the ImageNet dataset (https://www.image-net.org/challenges/LSVRC/2012/), surpassing other self-supervised methods and achieving high accuracy comparable to that of supervised baselines in the study [25]. In recent medical research, the BYOL has been employed in various fields. For instance, a study by Ren *et al*. [26] utilized BYOL for skin cancer diagnosis in teledermatology by applying Generative Adversarial Network based data augmentation with SSL. The generated augmented skin cancer images were trained using SimCLR and BYOL with the ResNet18 encoder. The SSL-trained models were then attached to a linear classifier for fine-tuning with labeled data to classify skin cancer images. Results from their proposed method with SSL demonstrated superior classification compared to those without SSL pre-training. The proposed BYOL achieved the highest accuracy (74.44%), surpassing SimCLR (72.5%) and SL (67.9%), highlighting the improvement of skin cancer classification through the use of the BYOL. In another study conducted by Taleb *et al*. [27], three SSL algorithms, including BYOL, were investigated for detecting tooth caries in bitewing X-ray images. This study demonstrated that using representation from SSL pre-trained ResNet18 encoders, followed by fine-tuning with a complete training set, resulted in all SSL models achieving higher sensitivity and receiver operating characteristic-area under the curve (ROC-AUC) compared to the SL baseline. Notably, the BYOL exhibited a specificity degree comparable to that of the baseline. A recent study by Feng *et al*. [28] proposed a method to improve semi-SL by integrating self-supervised BYOL for medical image recognition across three distinct datasets. These datasets include Optical Coherence Tomography (OCT2017), COVID-19 X-ray films encompassing normal, positive, and pneumonia cases, and Kvasir, which contains images across various classes, such as anatomical landmarks, pathological findings, and endoscopic procedures. Their method involves an initial pre-training phase with a small dataset, followed by retraining with labeled and pseudo-labeled data generated from the unlabeled data predictions obtained in the fine-tuning step. Their experimental results demonstrate the effectiveness of the proposed method, outperforming existing semi-supervised approaches in terms of accuracy across all three datasets, achieving accuracies of 96.6% for October 2017, 98.7% for COVID-19 X-ray, and 97.6% for Kvasir. In addition, a previous study by Pinetsuksai *et al*. [29] highlighted the potential of BYOL with human helminthic ova, exhibiting superior performance with over 95% accuracy even when trained with only 10% labeled data compared to the SL model. The aforementioned studies emphasize the potential of SSL, particularly BYOL, which presents a more efficient and effective approach for medical image representation learning. Its superior performance makes it interesting to use in parasite detection.

In this study, we propose employing the BYOL self-supervised approach to develop a neural network algorithm for identifying zoonotic blood parasites from microscopic images, marking the initial application of the BYOL for parasite species classification. In addition, we investigated the minimum proportion of labeled data required in the fine-tuning phase. Integrating the BYOL is anticipated to decrease the workload and dependency on parasite species annotation, thereby simplifying the training of deep learning models and aiding active surveillance tasks, particularly in remote areas lacking expensive laboratory apparatus.

## Materials and Methods

### Ethical approval

This study used a public dataset from a previous publication [30], which included microscopic images of Giemsa-stained thin blood films infected with various *Trypanosom*a species. Therefore, this study did not require ethical approval.

### Study period and location

This study was conducted from October 2022 to December 2023 at King Mongkut’s Institute of Technology Ladkrabang, Thailand.

### Data acquisition and preparation

This study used a public dataset from a previous publication [30], which included microscopic images of Giemsa-stained thin blood films infected with various *Trypanosoma* species. These specimens comprised late-stage *T. brucei* and late-stage *T. cruzi*, and late, round, and stumpy forms of *T. evansi*. The samples were captured using a digital camera (Olympus DP21-SAL, Tokyo, Japan) with the oil immersion field of a light microscope (Olympus CX31) (Table-1).

**Table-1:** Sample size of image sets per class.

Class name	Number of datasets
*Babesia*	1173
*Leishmania*	2389
*Plasmodium*	843
*Toxoplasma*	4547
*Trichomonad*	5513
Late-stage *Trypanosoma brucei*	4895
Late-stage *Trypanosoma cruzi*	276
Late-stage *Trypanosoma evansi*	5318
Round-stage *T. evansi*	2652
Stumpy-stage *T. evansi*	333
Erythrocyte	5299
Leukocyte	456
Microfilaria	465
Total	33694

Although it is recognized that creating both thick and thin smears improves the detection of acute phase trypanosomiasis, as noted in blood specimen processing guidelines from the Centers for Disease Control and Prevention [14, 15], the Clinical Microbiology Procedures Handbook by Leber [31] suggests that thin smears are more effective for identifying *T. cruzi* and other species. This is because the morphology of these organisms can be distorted in thick films. Consequently, our study used images of thin blood films to ensure that the parasites maintained their normal morphology.

**Figure-1:**
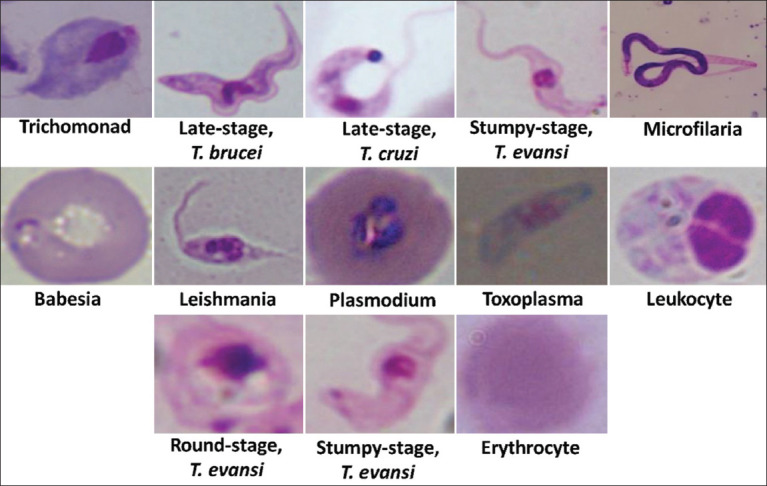
Thirteen class labels (nine species) used in this study.

The data detected in a prior study using YOLOv4-tiny on the CiRA CORE platform are publicly available from the GitHub repository at https://git.cira-lab.com/cira/cira-core. The original images were cropped to individual images before use in this study. In addition, we incorporated the microscopic images of parasite species from a public source [32], which included labeled non-trypanosome classes such as *Babesia, Leishmania, Plasmodium, Toxoplasma*, and *Trichomonad*, as well as both white and red blood cells. Another non-public dataset of microfilaria species was obtained from the Roboflow webserver (https://universe.roboflow.com/cdac-4hzts/microfilaria/dataset/1), which was cropped, augmented, and added to ensure the comprehensiveness of the study. Therefore, this study’s main dataset comprised a total of nine parasites and two blood cell types (Figure-1).

Both public datasets were divided into training, validation, and testing sets for image classification. The training set was mixed across classes for self-supervised pre-training. The entire training dataset was also fragmented into differently labeled percentages: 100%, 80%, 60%, 40%, 20%, 10%, 5%, and 1%. These partitioning and fractioning processes were selected randomly to minimize biases. In addition, individual images were resized to 224 × 224 pixels to match the input configuration required for the ResNet neural network.

### Architectural workflow

**Figure-2:**
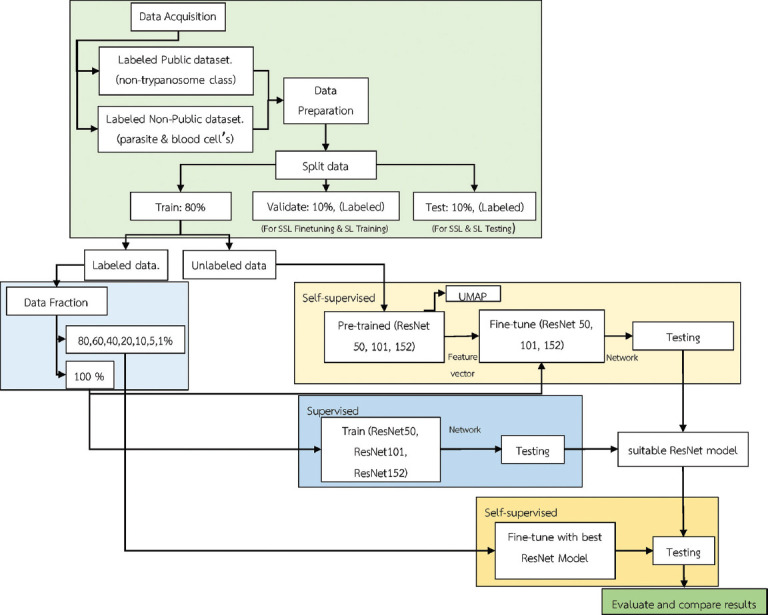
Study workflow.

The primary focus of model training in this study was to use the BYOL method of SSL to develop the network, with SL as a baseline for comparison (Figure-2). Initially, three versions of the ResNet model; ResNet50, ResNet101, and ResNet152 were employed as the backbone for both self-supervised and SL approaches using 100% labeled data. This step identified the most effective ResNet version, which was later fine-tuned to a fraction of labeled data for evaluation and comparison. The ResNet algorithms feature a unique architecture to avoid the vanishing gradient problem commonly encountered in deep networks. ResNet stabilizes training, mitigates the vanishing gradient problem, and enhances network performance by integrating batch normalization and shortcut connections between every two convolutional layers. This makes it particularly suitable for tasks such as parasite classification [33, 34].

Contrastive loss is a crucial component of BYOL [35]. The proposed method encourages the online network to produce similar representations for the same image under different augmentations (equation [1]). Simultaneously, it encouraged the online network’s embeddings to differ from those of other images. This method allows the model to learn meaningful and invariant representations from unlabeled data without relying on negative pairs.


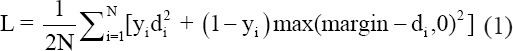


Here, N is the number of pairs, y_i_ is a binary label indicating whether the pair is similar (1) or dissimilar (0), d_i_ is the Euclidean distance between the embeddings of the two items in the pair, and margin is a margin parameter that defines the radius around the embeddings within which dissimilar pairs are not penalized.

For the SSL method, a pre-training step on unlabeled data was performed to generate feature vectors. These pre-trained weights were then fine-tuned using varying amounts of labeled data for downstream tasks. Uniform manifold approximation and projection (UMAP) was applied to the feature vectors before fine-tuning to visualize class grouping trends and understand the clustering of data points. This allowed us to observe how well the self-supervised method enabled the model to learn meaningful representations. If the data points of a class formed compact clusters and were well-separated from other classes, this indicated that the pre-trained weight would likely perform excellently in downstream tasks. The proposed method was only used with 100% labeled data for SL. All training processes, including pre-training and downstream classification, were conducted in a user interface-developed training configuration.

### Training configuration

Table-2 outlines the training conditions used in this study. The user interface of SSL allows users to modify all these parameters. For self-supervised pre-training with unlabeled data, a batch size of 128 and a learning rate of 0.125 were used, with a maximum of 6000 epochs. For supervised training and self-supervised fine-tuning, identical settings were applied, including a batch size of 128, a learning rate of 0.01, and a maximum of 500 epochs.

**Table-2:** Training configuration.

Model parameters	Self-supervised learning		Supervised learning

	Pre-train	Fine-tune	
Backbone	ResNets 50, 101, and 152		
Data label	No	Yes (With best ResNet version)	Yes (100%)
Batch size	128		
Learning rate	0.125	0.01	
Max epoch	6000	500	

**Figure-3:**
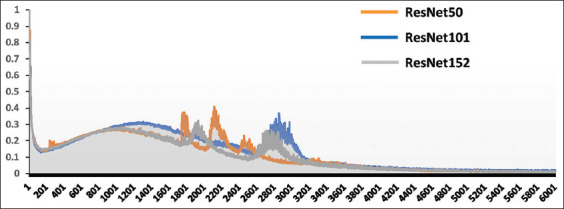
Historical loss of the pre-training process for ResNet50, ResNet101, and ResNet152. Each model underwent pre-trained 6000 epochs.

The training loss curve was monitored during self-supervised pre-training over a maximum of 6000 epochs (Figure-3). Initially, loss across all ResNet models increased in the first 1000 epochs before decreasing. However, a spike in the loss curve occurred between 1700 and 3000 epochs, indicating that the models had not yet reached saturation in their training during this period. Following this fluctuation, the loss curve for each model gradually declined and began to stabilize. The optimal training conditions were indicated by reaching a consistent minimum loss state for each model between 5000 and 6000 epochs.

### Evaluation of model performance

This study evaluates the network performance using a confusion matrix, which is a valuable tool for assessing the statistical significance of classification models. It examines metrics such as accuracy, recall, precision, F1 score, and AUC-ROC [18, 36, 37]. The confusion matrix visually represents the model’s performance by presenting four variables: true positive (TP), the prediction of a positive that was actually positive; true negative (TN), the prediction of a negative that was actually negative; false positive (FP), the prediction of a positive that was actually negative; and false negative (FN), the prediction of a negative that was actually positive. These statistical metrics can be expressed from TP, TN, FP, and FN as follows:

- Accuracy: This metric measures the overall correctness of the predictions made by the model, as expressed in equation (2). This indicates how effectively the model classified the given dataset. However, accuracy alone might be deceptive in scenarios of class imbalance because it can reflect the prevalence of the majority class rather than the actual prediction performance of the model.
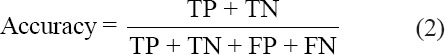
- Precision: This metric measures the accuracy of positive predictions (Eq. (3). This represents the ratio of TPs (correctly predicted positive observations) to the total number of predicted positives (sum of TP and FP).
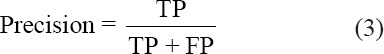
- Recall or Sensitivity (TP rate): This metric measures a model’s capability to identify all instances of the target classes, as expressed in equation (4). The ratio of TPs to total actual positives (sum of TP and FN).
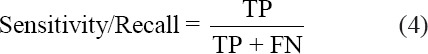

These metrics are particularly important for imbalanced datasets. Given that each class contains varying amounts of data, evaluating precision and recall aids in comprehending how effectively the model identifies and categorizes each specific class, especially those that are underrepresented.

- Specificity (TN rate): This metric measures a model’s capacity to correctly identify negative cases, as depicted in equation (5). It is significant when the absence of the condition is also a concern. It is defined as the ratio of TN to the sum of TN and FP.
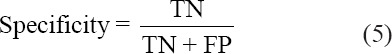
- F1 Score: This metric, calculated as the harmonic mean of precision and recall according to equation (6), offers a balanced measure between the two metrics. It is important to assess a model’s performance, particularly on a dataset where certain classes are important but less frequent.
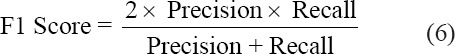

Furthermore, this study involved multi-class classification with imbalanced data; thus, macro averaging methods were employed for these metrics. This approach ensures that all classes are treated equally, which facilitates the detection of less frequent and more frequent classes for final evaluation [38].

Another evaluation metric utilized in this study was the AUC-ROC. The ROC curve plots the false positive rate (FPR, calculated as 1-specificity) on the X-axis against the TP rate (Recall) on the Y-axis at various decision thresholds (the probability that an instance is classified as positive). A higher AUC (close to 1) indicates superior performance [37].

## Results

### Development of a pre-trained BYOL (ResNet algorithms) model

**Figure-4:**
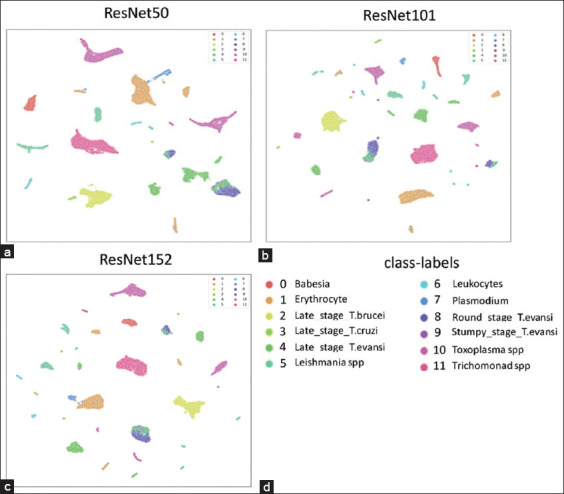
UMAPs. The UMAPs of (a) ResNet50, (b) ResNet101, and (c) ResNet152. (d) Class labels are represented by different colored dots. Clustering analysis of data points illustrates the similarity of individual extracted features. UMAPs=Uniform manifold approximation and projection.

The primary goal of the first result section was to find the best pre-trained weight using BYOL algorithms with three ResNet neural network backbones: ResNet50, ResNet101, and ResNet152 (Figures-4a–d). The UMAP visualizations of the three ResNet models pre-trained on unlabeled training datasets were examined. Data point clustering was more compact for ResNet101 and ResNet152 compared to ResNet50. However, the UMAP representations of ResNet50 were less dispersed than those of the larger models. This suggests that the ResNet50 model captures data patterns subtly, potentially identifying more detail than necessary for effective training and classification. In addition, UMAP analysis revealed clustering of *T. evansi* classes, including the round stage (dark blue), stumpy stage (violet), and a mix of the late stage (a second shade of green). This clustering indicates morphological similarity among the different stages of *T. evansi*, possibly due to the data being sourced from various animal species and the precise morphological characteristics of *T. evansi* in each species not yet being thoroughly identified.

### Supervised versus SSL models

**Figure-5:**
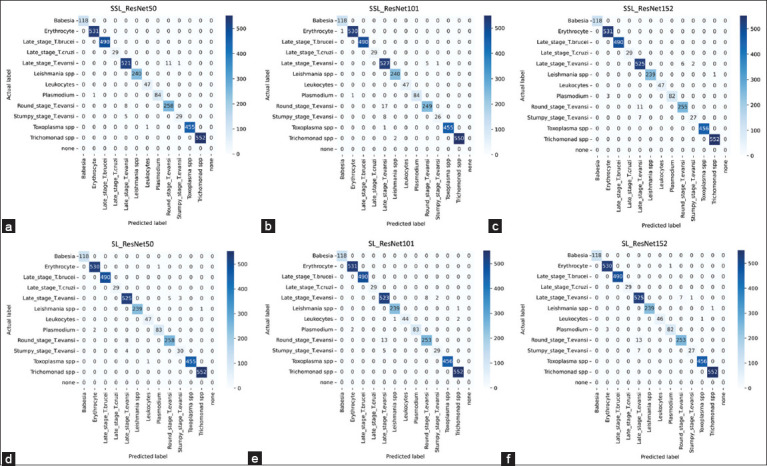
Confusion matrix table. Detailed confusion matrix tables of self-supervised learning algorithms: (a) ResNet50, (b) ResNet101, and (c) ResNet152. In addition, detailed confusion matrix tables for the supervised learning algorithms: (d) ResNet50, (e) ResNet101, and (f) ResNet152, all trained with 100% labeled data across the 12 classes.

The results section presents the confusion matrix table (Figures-5a–f), which shows the results of the supervised and self-supervised approaches trained with 12 classes of parasite data, each with 100% labeled training. Actual labels are on the Y-axis, and prediction labels are on the X-axis. The diagonal cells in the table indicate the correct classification for each class.

In particular, late-stage *T. evansi* classification performed best under SSL with ResNet101, obtaining 527 correct predictions out of 533 images. This was followed closely by SL with ResNet50 and both SL and SSL with ResNet152, each with 525 correct predictions. SL with ResNet101 correctly classified 523 images, and SSL with ResNet50 correctly classified 521 images. In this class, the highest score was obtained using the SSL approach. In addition, we observed that the misclassification of late-stage *T. evansi* in every confusion matrix is typically predicted as the early stages of *T. evansi*, namely, the round and stumpy stages, with a higher false prediction rate for the round stage than for the stumpy stage.

SL and SSL with ResNet50 achieved the highest number of correct predictions for the round stage, with 258 out of 266. SSL with ResNet152 obtained 255 correct classifications, and SL with both ResNet101 and ResNet152 each obtained 253 correct predictions. Next, SSL with ResNet101 obtained 249 correct predictions. For the stumpy stage, SL with ResNet50 obtained 30 out of 34 correct predictions, followed by SSL with ResNet50 and SL with ResNet101, each achieving 29 correct classifications. SL and SSL with ResNet152 correctly classified 27 images, and SSL with ResNet101 performed 26 accurate classifications. Finally, SSL with ResNet101 performed 26 accurate classifications. Misclassification from the actual round and stumpy-stage data was classified as the late stage of *T. evansi*, not as another early-stage class of the same species. In addition, all data from subclasses of *T. evansi* were correctly predicted within these subclasses, with no misclassifications of different Trypanosome species. This precision suggests that the models effectively distinguished *T. evansi* from other Trypanosomes. These findings align well with the patterns observed in the abovementioned pre-trained UMAP representations.

For the remaining parasite classes, all models successfully classified all test sets for *Babesia*, late-stage *T. brucei*, and *T. cruzi*. For erythrocytes, the best performance with all predictions correct was achieved by SSL with ResNet50 and ResNet152, as well as SL with ResNet101. Only one image was misclassified by the other models. For the *Leishmania* test set, SSL with ResNet50 and ResNet101 successfully classified all 240 images, while the remaining SL model and SSL with ResNet152 achieved 239 correct classifications. All models trained using the SSL approach and SL with ResNet50 correctly identified all 47 images for leukocyte detection. SL with ResNet101 and ResNet152 slightly underperformed, with 44 and 46 correct classifications, respectively. Both ResNet50 and ResNet101 trained under SSL correctly predicted 84 of 85 images for plasmodium. SL with ResNet50 and ResNet101 closely followed with 83 correct predictions, and SL with ResNet152 correctly identified 82 images. For Toxoplasma, all 456 images were correctly identified using SL with ResNet101, ResNet152, and SSL with ResNet152. The remaining models correctly predicted 455 out of the 456 images. Finally, for the *Trichomonad* class, all models correctly predicted all 552 images except for SSL with ResNet101, which correctly predicted 550 images.

Overall, the BYOL SSL models outperformed the SL models across all classes. Among all models trained using SSL, ResNet50 consistently achieved high accuracy in most classes.

### Evaluation of model performance

The results in this section presented evaluation metrics, including accuracy, recall (sensitivity), precision, and F1 score, derived from the preceding confusion matrix (Table-3). For SL, ResNet50 achieved the highest accuracy, recall, and F1-score scores (0.992606, 0.983791, and 0.984755, respectively. The highest precision for SL was observed with ResNet152 at 0.989916. In SSL, ResNet50 achieved the highest values for all the metrics: Accuracy of 0.992014, recall of 0.9822, precision of 0.989893, and F1-score of 0.98735. When trained with 100% labeled data, the SL and SSL performance metrics demonstrated similar values. SL scores were slightly higher in accuracy and recall, whereas SSL excels in precision and F1-score. These top values are highlighted in bold red. Although the differences in each metric are marginal, ResNet50 frequently obtained the highest results across most metrics. Consequently, ResNet50 was identified as the most suitable model for further training with fractionally labeled data. In addition, based on previous results, we considered the round and stumpy stage of *T. evansi* as an early stage, as we can differentiate between the early and late stages, which are crucial for indicating the severity of the infection.

**Table-3:** Comparison of evaluation metrics of SL and SSL trained with 100% labeled data. Bold values indicate the highest value for each statistical metric analyzed.

Evaluation metric (Macro-averaging)	Model	Value-based total data (100% labels)	

		Self-supervised learning	Supervised learning
Accuracy	ResNet50	0.992014	**0.992606**
	ResNet101	0.989352	0.989944
	ResNet152	0.991127	0.989944
Recall	ResNet50	0.9822	**0.983791**
	ResNet101	0.972506	0.974482
	ResNet152	0.974858	0.972301
Precision	ResNet50	**0.989893**	0.985786
	ResNet101	0.989809	0.988186
	ResNet152	0.988956	0.989916
F1-score	ResNet50	**0.98735**	0.984755
	ResNet101	0.980134	0.981063
	ResNet152	0.981412	0.98038

Bold values indicate the highest value for each statistical metric analyzed.

### Optimal model for a fractioned dataset with downstream processing

In the section on fractionally labeled data, we introduced microfilaria, expanding the scope of our study to include another flagellate parasite species. As a result, the total number of classes in the final step was 12. The microfilaria class underwent fine-tuning during the SSL step and was subsequently re-trained during the SL process for comparative analysis.

**Figure-6:**
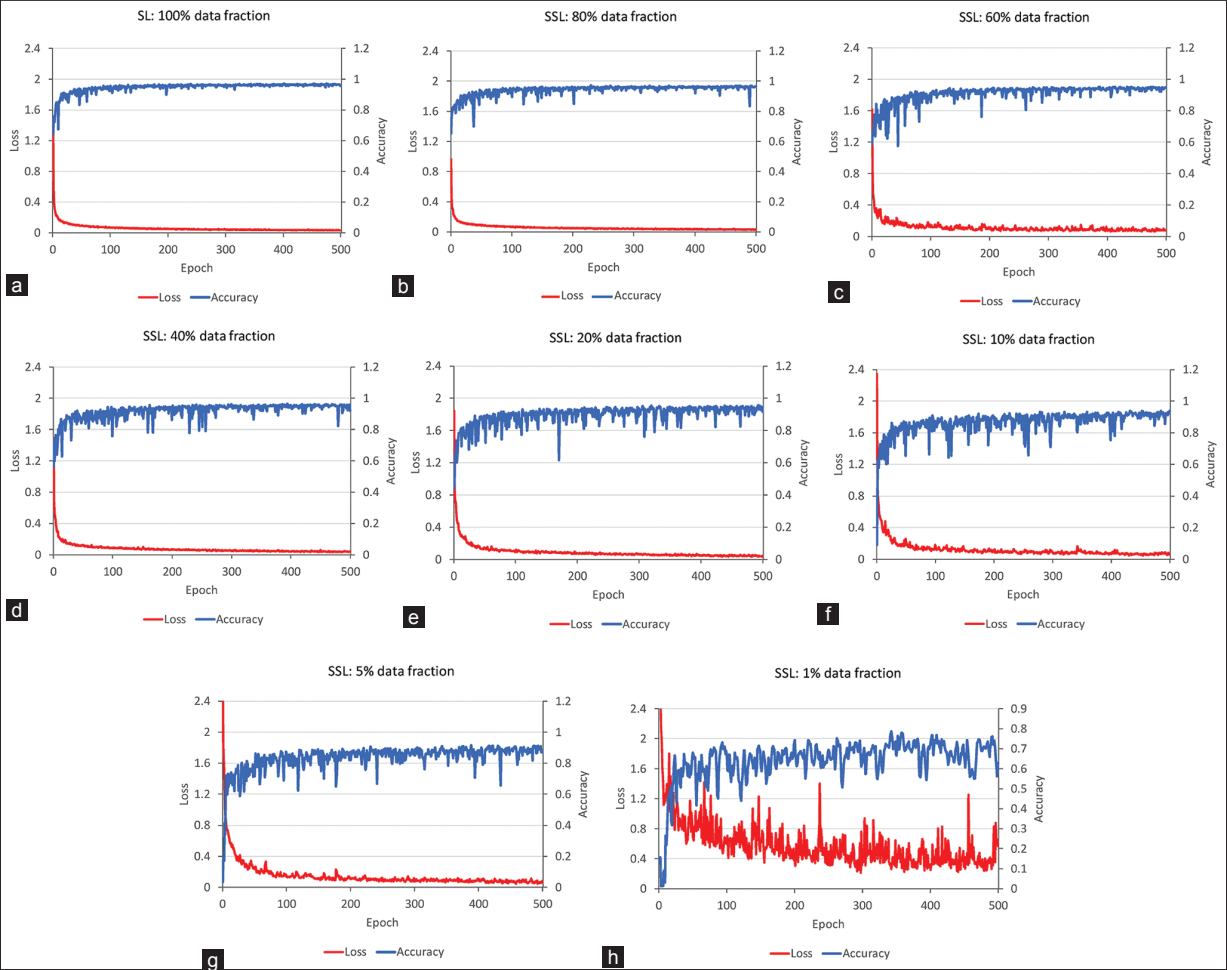
Training accuracy and training loss for (a) supervised learning algorithm and self-supervised learning algorithms fine-tuned with data fractions of (b) 80%, (c) 60%, (d) 40%, (e) 20%, (f) 10%, (g) 5%, and (h) 1% over 500 epochs.

Figures-6a–h show the training accuracy and loss curves obtained by SL training and SSL fine-tuning over 500 epochs. SL and SSL with 80% labeled data exhibit similar trends: an initial sharp decrease in loss that stabilizes at higher epochs, accompanied by a rapid increase in accuracy. This indicates that the model learned the data patterns effectively with the current configuration. Both scenarios also demonstrated plateaued accuracy at epoch 500, suggesting that training reached its optimal point. The final training loss for both scenarios was nearly identical, but the final accuracy was slightly higher for SSL at 80%. In subsequent training curves with varying percentages of labeled data, the final accuracy and loss follow a pattern in which accuracy decreases, and loss increases as the amount of labeled data decreases. This reflects the impact of the reduced labeled data on model performance. The fluctuations in the curves increased as the number of labeled data decreased. The trends for SSL with 60% and 40% labeled data remain stable; noise in the data decreases throughout training, which indicates that the current learning rate is effective. At epoch 500, the curves begin to level off, suggesting that extending training for an additional 200–300 epochs could lead to a more stable stage.

Conversely, the training curves for 20%, 10%, and 5% labeled data demonstrate less noticeable decreases in loss and increases in accuracy compared to scenarios with higher levels of labeled data. The significant noise observed in these curves suggests that the learning rate may be too high relative to the training duration. Adjusting the learning rate or increasing the number of training epochs improved the training outcomes. Finally, the SSL scenario with only 1% labeled data over 500 epochs exhibits considerable fluctuations without a clear reduction in loss or improvement in accuracy, which indicates that the current amount of data and training configuration are insufficient. Adjustments to parameters and model architecture may be required to enhance model performance.

### Evaluation of fine-tuning models on various fractioned data

**Figure-7:**
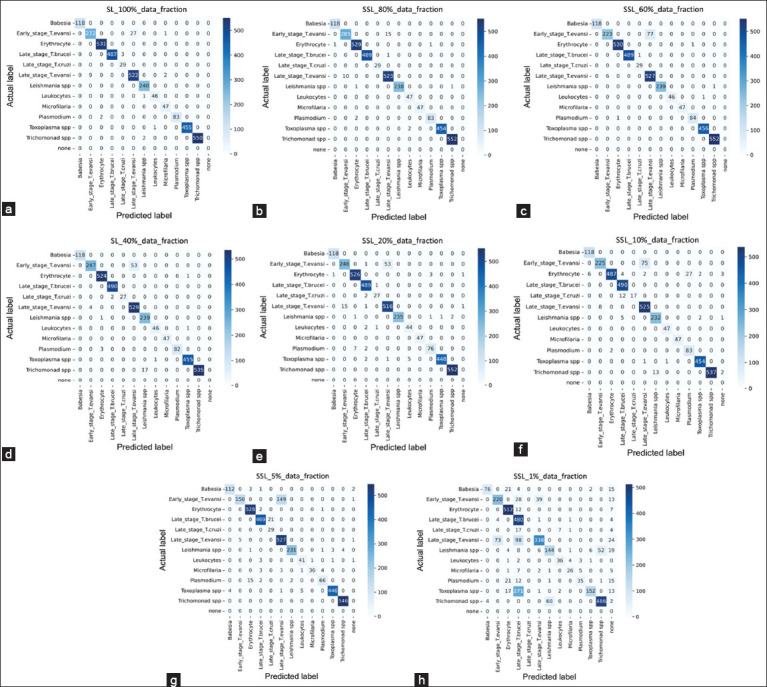
Confusion matrix tables of fine-tuned fractional labeled data, encompassing (a) supervised learning algorithm and self-supervised learning algorithms, with fractions of (b) 80%, (c) 60%, (d) 40%, (e) 20%, (f) 10%, (g) 5%, and (h) 1% over 500 epochs.

This study evaluated all models under identical configurations at epoch 500 to ensure fair comparison (Figures-7a–f). The confusion matrix for all scenarios is provided below:

When comparing the confusion matrix of SL-100% and SSL-80% after adding microfilaria and combining round- and stumpy-stage *T. evansi* as early stages, SSL-80% tended to provide more accurate predictions in most classes, including leukocytes, *Trichomonad*, late-stage *T. brucei*, and both early and late-stage *T. evansi*. SL-100% and SSL-80% correctly classified *Babesia*, late-state *T. cruzi*, Microfilaria, and Plasmodium with equal accuracy. However, SL-100% gains more true classifications in the remaining three classes than SSL-80%, often by more than one or two items. SL-100% and SSL-80%, when reduced to SSL-20% labeled data, can maintain correct classification rates exceeding 80% for every class. SSL-10% can achieve correct classification exceeding 80% for the test set of each class for 10 out of 12 classes, with the remaining two classes (early-stage *T. evansi* and late-stage *T. cruzi*) exceeding 70% of the total test set of that class. SSL-5% also achieves 80% correct classification for nine classes, while it exceeds 70% of the test set for two classes (Microfilaria and Plasmodium) and 60% for the remaining class (early-stage *T. evansi*). SSL with 1% labeled data reached over 80% accuracy for the three classes (Erythrocyte, late-stage *T. brucei*, and *Trichomonad*), over 70% for the two classes (early-stage *T. evansi*, and Leukocyte), and over 60% for the three classes (*Babesia*, late-stage *T. evansi*, and *Leishmania*). However, this model achieved only over 50%, 40%, and 30% accuracy for one class each – microfilaria, *Plasmodium*, and *Toxoplasma*, respectively and completely misclassifies all tests of late-stage *T. cruzi*.

As can be seen, a large amount of labeled data typically leads to higher accuracy across more classes. In contrast, a reduction in labeled data often results in increased misclassification, as indicated by the higher counts of off-diagonal cells in the confusion matrix.

Focusing on the classification of *T. evansi* species, the SL-100% model occasionally misclassified some *T. evansi* test sets as belonging to the Microfilaria class. In contrast, SSL models with 80%–10% labeled data did not misclassify *T. evansi* as any other trypanosome species. Specifically, SSL models with 80%–40% and 10% labeled data contained misclassifications only within the *T. evansi* species, indicating that these models can differentiate between sub-species of *Trypanosoma*. This observation highlights the effectiveness of pre-trained weights in SSL, which appear to contribute to accurate classification even when training data are not fully labeled. In comparison, the SL-100% model, despite being trained on a complete dataset, still showed errors in *T. evansi* classification. This pre-training on partial data suggests that SSL can better capture the distinct features necessary for accurate species classification.

**Figure-8:**
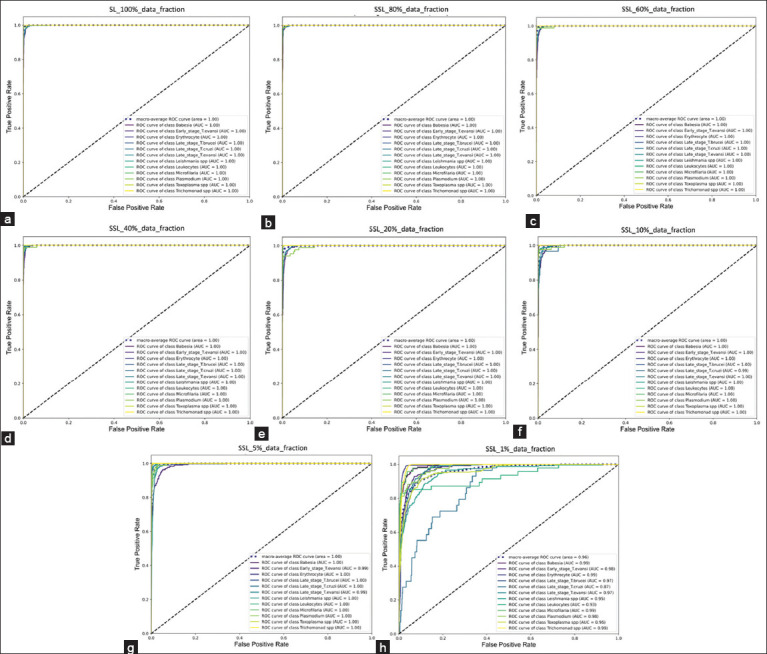
Receiver operating characteristics-area under the curve values for supervised learning and self-supervised learning algorithms, including (a) supervised learning fine-tuned with total data and (b)–(h) self-supervised learning fine-tuned with 80%, 60%, 40%, 20%, 10%, 5%, and 1% fractional labeled data, respectively.

Figure-8 illustrates the ROC curves and their corresponding AUC values for various classes, where each class is represented by a different color as indicated in the legend located at the bottom right corner of each chart. The closer the curves are to the top left corner of the graph and the closer the AUC value is to 1.0, the better the model’s classification accuracy across all thresholds (indicating less sensitivity to variations in the threshold that determines whether a prediction is classified as positive or negative). The table above shows that most clusters are positioned at the top left corner, ranging from SSL 80%, SL 100%, SSL 60% to 1%. The fewer labeled data were available, the more noticeable the downward shift of the curve in multiple classes was, resulting in AUC values slightly <1.00. In particular, for SSL-1%, there was a significant decrease in both the proximity of the ROC curve to the top left corner and the AUC values (0.96), indicating a decrease in performance compared to the other models. However, an AUC value of 0.96 is still considered excellent, reflecting the high level of model capability, even with only 1% supervision.

In the ROC space, a diagonal line represents a no-skill classifier that correctly predicts outcomes 50% of the time. The positioning of all model curves above this line confirms that each model outperformed the random guess.

Analyzing all evaluation metrics shown in Table-4, both the SL-100% model and SSL models with fractional labeled data from 80% to 10% achieved >90% across all metrics. This performance trend is consistent with the findings from the confusion matrix and ROC-AUC results, where the score of each metric decreases as the percentage of labeled data.

**Table-4:** Evaluation metrics for SL-100% and SSL with fractional labeled data are provided. SL and SSL denote supervised and SSL, respectively. The macro-averaged values were evaluated from the best-selected model obtained after training for 500 epochs.

Metric	SL	SSL						
	
	100%	80%	60%	40%	20%	10%	5%	1%
Accuracy	0.9977	**0.9983**	0.9957	0.9957	0.9950	0.9921	0.9881	0.9581
Recall	0.9858	**0.9908**	0.9742	0.9700	0.9583	0.9292	0.8945	0.6250
Precision	0.9773	**0.9890**	0.9829	0.9768	0.9648	0.9512	0.9119	0.7424
Specification	0.9987	**0.9991**	0.9975	0.9975	0.9971	0.9956	0.9932	0.9775
F1-score	0.9812	**0.9898**	0.9770	0.9724	0.9610	0.9328	0.8897	0.7370
Area under the curve	**1.0000**	**1.0000**	**1.0000**	**10000**	**1.0000**	**1.0000**	**1.0000**	0.9600

SL=Supervised learning, SSL=Self-supervised learning Bold values indicate the highest value for each statistical metric analyzed

Interestingly, SSL with 80% labeled data demonstrated the highest performance across all metrics, achieving an accuracy of 0.9983, recall of 0.9908, precision of 0.9890, specificity of 0.9991, and an F1-score of 0.9898. This performance level surpasses that of SL with fully labeled data and correlates with the more tightly clustered AUC curve for SSL-80% discussed earlier. This superior performance of SSL-80%, along with the fact that even a reduced amount of volume data to as little as 10% can still achieve over 90% accuracy, approaching that of the SL approach, highlights the superiority of SSL techniques over the SL model and demonstrates their effectiveness for parasite classification.

### Class-wise comparison

We further analyzed the accuracy and recall metrics for each class (crucial for the screening application) across all SSL models to determine the minimum percentage required for SSL training to fulfill the purpose of this study. The SSL model with 20% labeled data demonstrated the lowest percentage that achieved all metrics >80% for the early *T. evansi* class and >90% for the late-stage *T. evansi* class (Table-5).

**Table-5:** Performance of the self-supervised learning -20% model by class labels.

Class name	Accuracy	Recall	Precision	Specificity	F1-score
*Babesia*	0.9997	1.0000	0.9916	0.9997	0.9958
Early-stage *Trypanosoma evansi*	0.9799	0.8200	0.9425	0.9952	0.8770
Erythrocyte	0.9962	0.9906	0.9850	0.9972	0.9878
Late-stage *Trypanosoma brucei*	0.9968	0.9980	0.9800	0.9966	0.9889
Late-stage *Trypanosoma cruzi*	0.9988	0.9310	0.9310	0.9994	0.9310
Late-stage *T. evansi*	0.9793	0.9681	0.9053	0.9813	0.9356
*Leishmania* spp.	0.9985	0.9792	1.0000	1.0000	0.9895
Leukocytes	0.9977	0.9362	0.8980	0.9985	0.9167
Microfilaria	1.0000	1.0000	1.0000	1.0000	1.0000
*Plasmodium*	0.9962	0.8941	0.9500	0.9988	0.9212
*Toxoplasma* spp.	0.9974	0.9825	0.9978	0.9997	0.9901
*Trichomonad*	0.9994	1.0000	0.9964	0.9993	0.9982

The classes demonstrated performance that exceeded 90% for all evaluation metrics, while the remaining 10 classes achieved this level of performance. Table-5 provides a detailed overview of the performance. Based on these findings, we conclude that the minimum percentage required to train SSL with hemoprotozoan parasite species is 20%.

The outstanding performance of SSL in this study can be attributed to several key factors. First, the unique characteristics of the parasite species play a role, as does the BYOL algorithm, which can extract and differentiate crucial features from the dataset. This enables the effective mapping of pre-train weight to specific classes through a fine-tuning process, resulting in a final high-performance model. In addition, the preprocessing step involving the YOLOv4-tiny neural network aids in accurately cropping multicellular parasites [30], thus providing precise boundaries of the cell and simplifying feature extraction from the dataset. Furthermore, using thin-film smear techniques to prepare trypanosome species samples ensures that accurate morphology is maintained, thereby enhancing the effectiveness of the model training. Collectively, these elements contributed to the achievement of superior performance in this study.

## Discussion

This study addressed the challenge of detecting zoonotic blood parasites, which pose significant health risks in various regions, including Thailand. Precisely diagnosing these parasites is crucial for preventing their spread and effectively managing outbreaks, particularly in regions with limited diagnostic resources or expertise.

We have introduced BYOL, a novel SSL method, to develop a classification model for microscopic images. This study involved training on nine species of parasites and blood cells, namely *Babesia, Leishmania, Plasmodium, Toxoplasma, Trichomonad*, erythrocyte, leukocyte, late-stage *T. brucei*, late-stage *T. cruzi*, and both late-stage and early-stage (round and stumpy morphology) *T. evansi*, using ResNet models. Initially, training used 100% of the labeled datasets, employing ResNet50, ResNet101, and ResNet152 models through supervised and BYOL’s SSL approach. The BYOL algorithm incorporates components such as the online and target networks, enabling feature extraction without class labeling by minimizing the loss of similarity between networks to optimize weights [25]. Subsequently, this pre-trained weight undergoes fine-tuning with multi-class labeling.

The preliminary results demonstrate that ResNet50 trained through SSL outperformed the SL approach, indicating that ResNet50 is suitable for the complexity of this dataset and offers an advantage in reducing training time due to the fewer layers. We further fine-tuned ResNet50 with pre-trained SSL weights with different proportions of labeled data over 500 epochs – 80%, 60%, 40%, 20%, 10%, 5%, and 1% – and compared it to the SL method. With 80% labeled data, the BYOL model outperformed fully supervised models. Intriguingly, with only 20% labeled data, the model achieved a performance comparable to that of the SL method, with overall evaluation metrics (accuracy, recall, specificity, precision, and F1-score) exceeding 90% and a ROC-AUC of 1. In addition, it achieved over 80% for the specific detection of early-stage *T. evansi* (accuracy of 98% and recall of 82%) and over 90% for late-stage *T. evansi* (with an accuracy of 98% and recall of 97%). This demonstrates that the model developed using BYOL’s SSL can effectively distinguish different morphologies and differentiate them from other species with high accuracy, even with a limited number of labeled datasets. This underscores the potential superiority of BYOL’s SSL and demonstrates that this enhanced method offers a more practical solution than state-of-the-art deep learning methods that heavily rely on extensive classes provided by experts.

In addition, BYOL exhibits an advantage in handling variations in data, such as differences in color staining and cell placement within images. This is because the BYOL workflow incorporates several data augmentation techniques within its architecture, enabling it to generate diverse representations of an image. This feature particularly benefits real-life data, where sample preparation methods can vary.

In Table-6, we compare our approach to other studies [16, 20] that do not use fully labeled data to classify parasite species. One such study used an SSGL technique for *Plasmodium, Babesia*, and *Toxoplasma* [16]. Our model, which leverages pre-trained weights from BYOL, demonstrates superior performance compared to other semi-supervised models in this study and SSGL, particularly when accuracy is calculated only on matched classes and evaluation metrics with 20% labeled data. Another study by Ren *et al*. [20] focused on training apicomplexan species by employing contrastive learning between weakly labeled microdata and the corresponding labeled macro data, with an annotation percentage of 1%. Our proposed method surpasses this approach by 1.4% accuracy, as shown in Table-6, when accuracy is calculated only for matched classes with 1% labeled data. These findings highlight the efficiency of BYOL for parasite classification. Moreover, our study represents the first attempt to experiment with multiple hemoprotozoan parasites, especially zoonotic species, in conjunction with a self-supervised approach.

**Table-6:** Comparison of the accuracy of the BYOL approach for single cell-apicomplexans classification with another semi-supervised study at an equivalent fraction of labeled data.

Method	Accuracy	Reference
Pi-model	77.42	[16]
Mix-Match	88.25	[16]
Semi-supervised graph learning	91.75	[16]
BYOL of 20%	99.73	[Our study]
Micro- and macro-data	94.90	[20]
BYOL with 1%	96.34	[Our study]

BYOL=Bootstrap your own latent

The impressive outcomes achieved in this study can be attributed to the meticulous data preparation undertaken before training. This process involves cropping multi-class images into single-cell images using the YOLOv4-tiny model [39, 40], which helps to precisely delineate cell boundaries and enhances feature extraction accuracy. Therefore, this model may not be applicable to microscopic images of the entire field. In addition, specimen preparation involved using non-public data methods to maintain the actual morphology of the parasite using thin smears. Although SSL allows for the use of smaller labeled datasets during training, it still requires datasets containing regions of significant variability for effective optimization [41].

However, this limitation can be alleviated by incorporating additional data augmentation techniques before training. Furthermore, SSL training requires substantial computational resources to train with large datasets and batch sizes (which were set at 128 in this study), resulting in longer time consumption than traditional SL because the pre-trained step must be completed initially. However, training parameters can be adjusted to reach the optimal computer limit, and any increase in training time is offset by a reduction in the time required to label the data. Therefore, SSL is suitable for scenarios where labeling may be challenging or when significant data variations exist from different observers. In summary, employing a smaller amount of labeled data requires a longer training duration; however, using a larger amount of labeled data reduces training time. This observation is supported by the training and loss curves, which demonstrate that a lower percentage of labeled data requires more fine-tuning time to realize a stable training stage.

## Conclusion

The model trained using the BYOL SSL approach has proven efficient and successful in detecting zoonotic parasites. Moreover, its applicability extends to other species examined in this study, making it a comprehensive and potentially universal model for identifying coinfections. This could enhance disease control and mitigate efforts aimed at enhancing disease control and mitigating outbreaks, particularly in settings with limited resources. Finally, in addition to its efficacy in parasite detection, SSL addresses notable challenges such as data variability and the requirement for extensive class labeling, which are common in biology and medical fields. The integration of BYOL’s SSL facilitates the broader adoption of deep learning models in global health settings, thereby enhancing diagnostic and treatment efficacy.

## Authors’ Contributions

SBu and VK: Designed the study, project administration, and drafted the manuscript. SBu, MK, and VK: Methodology. MK: Investigation. SBu, NP, and VK: Formal analysis. NP, TT, SC, and SBo: Software and validation. MK and VK: Data curation. VK, SC, and SBo: Visualization. VK, MK, SC, and SBo: Drafted, reviewed, and edited the manuscript. All authors have read and approved the final version of the manuscript.
